# Invasive fungal disease in COVID-19 patients: a single-center prospective observational study

**DOI:** 10.3389/fmed.2023.1084666

**Published:** 2023-06-09

**Authors:** Tatjana Adzic-Vukicevic, Milos Mladenovic, Snezana Jovanovic, Ivan Soldatović, Aleksandra Radovanovic-Spurnic

**Affiliations:** ^1^Faculty of Medicine, University of Belgrade, Belgrade, Serbia; ^2^Covid Hospital Batajnica, University Clinical Center of Serbia, Belgrade, Serbia; ^3^Clinic for Pulmonology, University Clinical Center of Serbia, Belgrade, Serbia; ^4^Clinic for Infectious and Tropical Diseases, University Clinical Center of Serbia, Belgrade, Serbia; ^5^Institute for Medical Statistics and Informatics, Faculty of Medicine, University of Belgrade, Belgrade, Serbia; ^6^Center for Microbiology, University Clinical Center of Serbia, Belgrade, Serbia

**Keywords:** COVID-19, aspergillosis, candidiasis, treatment, study

## Abstract

**Background:**

Invasive fungal diseases (IFDs) are caused by fungal infections that manifest as serious secondary infections in patients with COVID-19. The increased morbidity and mortality rates are most frequently observed in patients with COVID-19-associated pulmonary aspergillosis (CAPA) and COVID-19-associated candidiasis (CAC). CAPA is the most frequently encountered infection with an incidence rate of 0.7–7.7%, while CAC is a less common and less studied fungal infection in COVID-19 patients.

**Materials and methods:**

The present article is a prospective observational single-center study that was conducted between 1 September 2021 and 24 December 2021, involving 6,335 patients who were admitted to COVID Hospital “Batajnica,” University Clinical Center of Serbia, Belgrade.

**Results:**

Of the 6,335 patients hospitalized during the four-month period of the study, 120 patients (1.86%) who had a proven diagnosis of IFD were included in the study. These patients were divided into two groups: CAPA patients (*n* = 63) and CAC patients (*n* = 56); however, one of the 120 patients was diagnosed with *Cryptoccocus neoformans* infection. The mean age of the study population was 65.7 ± 13.9 years, and 78 (65.5%) of them were men. The patients were identified to have the following non-malignant comorbidities: arterial hypertension in 62 (52.1%) patients, diabetes mellitus in 34 (28.65), pre-existing lung damage similar to that observed in COPD and asthma in 20 (16.8%), and chronic renal insufficiency in 13 (10.9%) patients. The hematological malignancies were found to be the most prevalent malignancies and were identified in 20 (16.8%) patients, particularly in CAPA patients [11 (17.5%); *p* < 0.041]. Fiberoptic bronchoscopy with bronchoalveolar lavage fluid (BALF) and microscopic examination confirmed the presence of fungal infections in 17 (14.3%) patients. Serology testing was also performed in the majority of cases. Antibodies against *Aspergillus* spp. and *Candida* spp. were predominantly found in CAPA patients (*p* < 0.001). The patients were also tested for the presence of (1–3)-β-D glucan (*p* < 0.019), galactomannan, and mannan in the specimens. Blood cultures were found to be positive in 45 (37.8%) patients, mostly in CAC patients. Mechanical ventilation was applied in 41 (34.5%) patients, while a non-invasive technique, such as continuous positive airway pressure (CPAP) or high-flow nasal cannula (HFNC), was used in 20 (16.8%) patients. The following antifungals were administered: echinocandins in 42 (35.3%), voriconazole in 30 (25.2%), and fluconazole in 27 (22.7%) patients. Most of the patients received systemic corticosteroids (mainly methylprednisolone), while 11 (9.16%) received favipiravir, 32 (26.67%) remdesivir, 8 (6.67%) casirivimab/imdevimab, and 5 (4.16%) sotrovimab. The outcome was lethal in 76 (63.9%) patients, predominantly CAC patients (*p* < 0.001).

**Conclusion:**

Invasive fungal disease is a severe complication associated with COVID-19 and accounts for increased mortality in these patients. Early identification and appropriate treatment may provide a favorable outcome.

## Introduction

Invasive fungal disease (IFD) is a serious secondary infection that affects patients with COVID-19 and acute respiratory distress syndrome (ARDS). It is associated with an increased mortality rate of 16–25% in COVID-19 and ARDS patients as compared to patients without evidence of fungal disease (1). A high mortality rate was also observed in patients with influenza-associated pulmonary aspergillosis (IAPA), and their survival rate was 24% lower than that observed in patients without secondary infection (2). The risk factors that contribute to the manifestation of IFD are still unclear. IFD might be caused by severe acute respiratory syndrome coronavirus 2 (SARS-CoV-2) itself or due to additional risk factors, such as corticosteroid or anti-interleukin (IL)-6 therapy with tocilizumab (3). Corticosteroid therapy in patients with severe COVID-19 showed survival benefits, as was reported in the RECOVERY trial (4). Furthermore, corticosteroid and anti-IL-6 treatment may result in increased susceptibility to bacterial and fungal super-infections (5).

Invasive fungal diseases (IFDs) that are commonly observed in COVID-19 patients include COVID-19-associated pulmonary aspergillosis (CAPA), COVID-19-associated candidiasis (CAC), coccidioidomycosis, fusariosis, histoplasmosis, mucormycosis, pneumocystosis, and saccharomycosis (6). CAPA is the most frequently reported disease in the literature with an incidence rate of 0.7–7.7% among patients with COVID-19 and of 2.5–39.1% among patients admitted to the intensive care unit (ICU) (7). Although CAC is a less common and less studied fungal infection in COVID-19 patients, it is more prevalent in some countries (8). Respiratory viruses such as SARS-CoV-2 cause severe damage to the airway epithelium and enable tissue invasion by fungal pathogens. Similar to other SARS coronaviruses, SARS-CoV-2 targets and invades epithelial cells and type II pneumocytes, binding SARS spike protein to the angiotensin–converting enzyme 2 (ACE2) receptors (9). IFD presents as the most severe manifestation of fungal infection that is associated with a high mortality rate. IFD causes serious complications in immunosuppressed patients, such as those with hematologic malignancies, as well as in those who receive systemic corticosteroids to treat structural lung damage like chronic obstructive pulmonary disease (COPD) (10). Other risk factors that contribute to the development of IFD in COVID-19 patients are liver cirrhosis, systemic connective tissue disease, chronic kidney disease or renal replacement therapy, influenza infection, diabetes mellitus, and advanced solid cancers (11). This study aimed to determine the clinical characteristics of the CAPA and CAC with the help of diagnostic procedures, clinical course, and treatment outcome.

## Materials and methods

A prospective observational single-center study was conducted between 1 September 2021 and 24 December 2021 involving 6,335 patients admitted to the COVID Hospital “Batajnica,” University Clinical Center of Serbia, Belgrade. The inclusion criteria were as follows: (1) patients presenting a positive result in the reverse transcription-polymerase chain reaction (RT-PCR) assay for SARS-CoV-2 in respiratory specimens (nasopharyngeal swab, tracheal aspirate, bronchial aspirate, or bronchoalveolar lavage fluid) and (2) patients suspected to have IFD based on clinical features, thoracic CT scan, culture, serology, and galactomannan, mannan, and (1-3)-β-D-glucan tests.

*Aspergillus* infections were classified as possible/probable/proven according to the European Organization for Research and Treatment of Cancer/Invasive Fungal Infections Cooperative Group and the National Institute of Allergy and Infectious Disease Mycoses Study Group (EORTC/MSG) (12). The diagnostic criteria for probable/proven fungal infections were as follows: (1) *Candida-* or *Aspergillus*-positive lower respiratory tract culture; (2) compatible signs and symptoms, such as fever refractory of at least 3 days of appropriate antibiotic therapy, dyspnea, hemoptysis, worsening respiratory insufficiency despite appropriate antibiotic therapy and ventilation support; (3) abnormal chest X-ray or CT scan; and (4) positive microbiological culture of *Candida* spp. or *Aspergillus* spp. in BALF without bacterial growth and positive cytological smear showing branching hyphae. Positive serology results indicating the presence of *Aspergillus* galactomannan (GM) or *Candida* mannan antigens and (1-3)-β-D glucan in BAL, sputum, and serum were added to the existing algorithm as the microbiological criteria for identification [modified Alternative algorithm adapted to the intensive care unit (ICU) for aspergillosis] (13).

### Microbiological analysis

Serological tests were performed to determine the presence of the following antigens and antibodies in the given specimen: (1–3)-β-D glucan (FUJIFILM Wako Pure Chemical Corporation, Osaka, Japan), *Aspergillus* galactomannan antigen (Dynamiker Aspergillus Galactomannan Assay, Tianjin, China), anti-*Aspergillus* IgM (TECAN, Hamburg Germany), anti-*Aspergillus* IgG (TECAN, Hamburg Germany), *Candida* mannan antigen (Dynamiker *Candida* Mannan Assay Tianjin, China), anti-*Candida* IgM (Nova Lisa Dietzenbach, Germany), and anti-*Candida* IgG (Nova Lisa Dietzenbach, Germany). *Aspergillus* spp. were identified by the cultivation on Sabouraud dextrose agar (SDA), phenotypic identification based on the appearance of colonies, wet mounts of the culture, and Riddell’s classic slide culture method. *Candida* spp. were identified by culturing the samples on a chromogenic medium CHROMID *Candida* agar (bioMerieux, France) and using the identification card VITEK 2 YST ID Card (bioMerieux France).

A real-time fluorescent RT-PCR kit designed by BGI (BGI Group, Shenzhen, China) was used to detect SARS-CoV-2.

**Figure 1:**
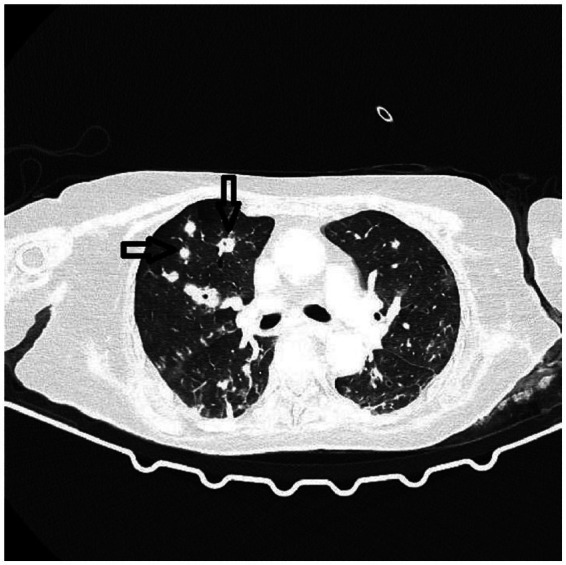
The CT scan shows dense, well-circumscribed lesions without a halo sign.

**Figure 2:**
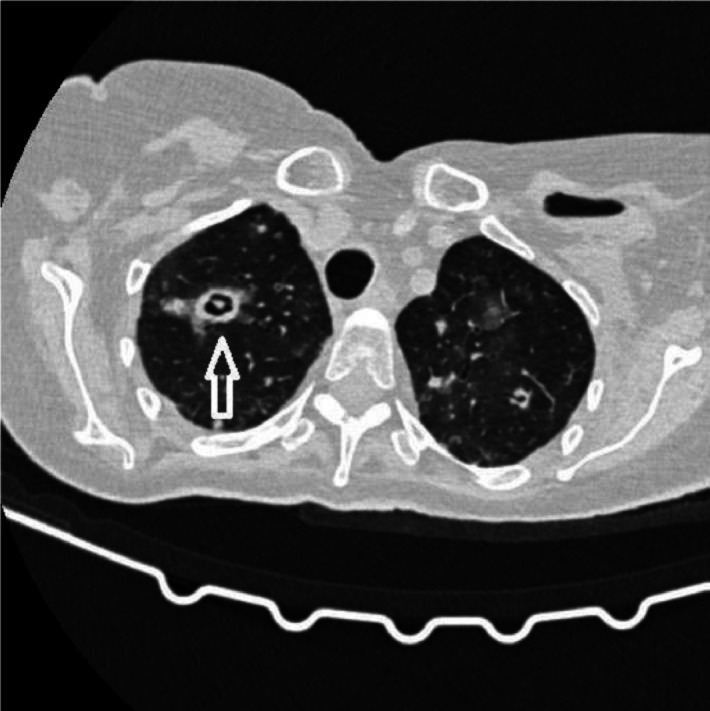
The CT scan shows an air crescent sign.

**Figure 3:**
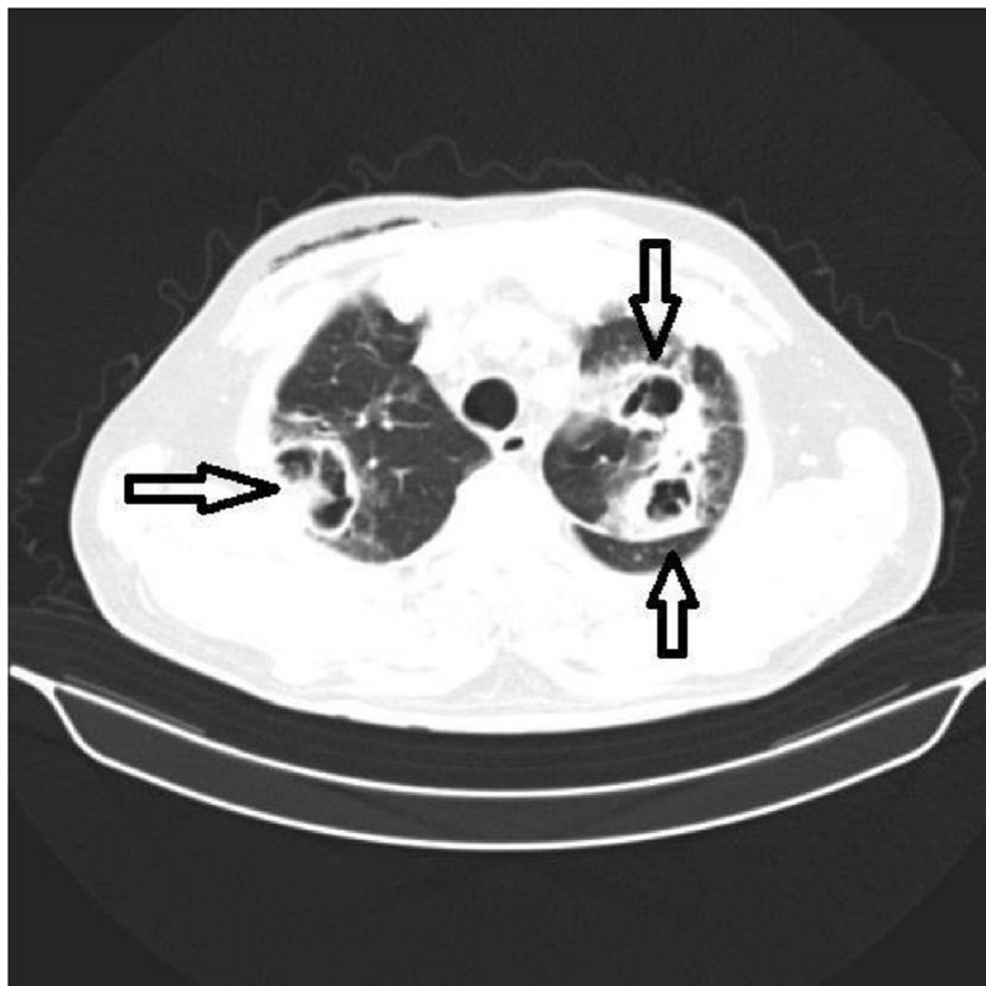
The CT scan shows cavitary lesions.

**Figure 4:**
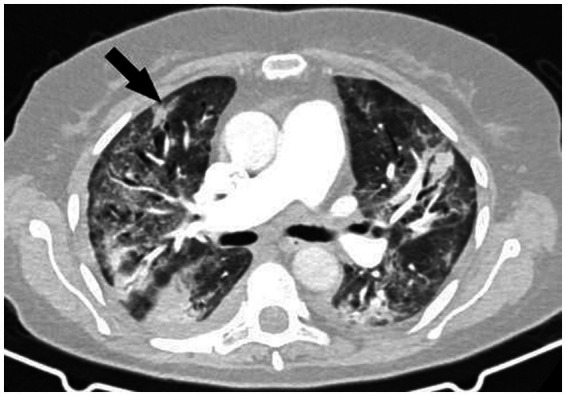
The CT scan shows wedge-shaped and segmental or lobar consolidation.

A thoracic CT scan was performed on all the patients. The cases were considered to be positive if one of the following four criteria was present: dense, well-circumscribed lesions with or without halo signs (Figure 1), an air crescent sign (Figure 2), cavitary lesions (Figure 3), and wedge-shaped and segmental or lobar consolidation (Figure 4).

The data regarding the demographic characteristics of patients and clinical outcomes, such as age, sex, comorbidities, and microbiological disease confirmation, were obtained from the electronic medical records of the patients.

### Statistical analysis

The results of the study are presented as numbers (%), means ± standard deviation, or median (25th–75th percentile) depending on the data type and distribution. The groups were compared using parametric (*t*-test) and non-parametric (chi-squared, Fisher’s exact test, and Mann–Whitney *U*-test) tests. All value of ps less than 0.05 were considered significant. The data were analyzed using SPSS 20.0 (IBM Corp. Released 2011, IBM SPSS Statistics for Windows, Version 20.0, Armonk, NY) and R 3.4.2 software [R Core Team (2017). R: A language and environment for statistical computing. R Foundation for Statistical Computing, Vienna, Austria].^1^

## Results

**Figure 5:**
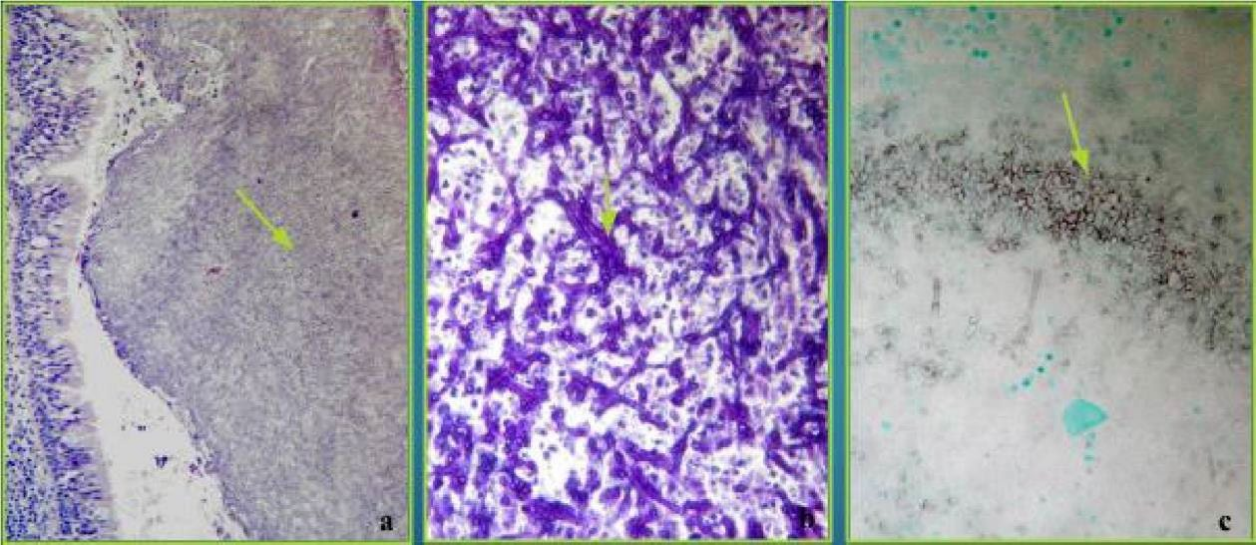
Histopathological findings (hematoxylin–eosin and Grocott-Gomori’s methenamine silver stain). **(A)** Vascular infiltration of hyphae with infarcts in lung parenchyma without inflammation, H&E x10. **(B)** Necrotic granuloma reaction, periodic acid–Schiff PAS x2. **(C)** Wide branching hyphae with spores, Gomori’s methenamine silver x10.

This study included 120 (1.86%) patients of the 6,335 hospitalized ones with a proven diagnosis of IFD during the four-month period. SARS-CoV-2 delta-driven surge was confirmed in all patients. These patients were divided into two groups according to the fungal species they were diagnosed to be infected with: CAPA patients (*n* = 63) and CAC patients (*n* = 56); however, one patient was diagnosed with *Cryptococcus neoformans* infection. The mean age of the study subjects was 65.7 ± 13.9 years, and 78 patients (65.5%) were men. Patients with CAPA were younger (61.4 ± 14.7 years; *p* < 0.001) than those with CAC (Table 1). The results of the laboratory test showed severe lymphopenia (*p* < 0.021) and increased IL-6 levels (*p* < 0.004) in CAPA patients (Table 2). The following non-malignant comorbidities were detected: arterial hypertension in 62 (52.1%), diabetes mellitus in 34 (28.65), respiratory disorders such as COPD and asthma in 20 (16.8%), and chronic renal insufficiency in 13 (10.9%) patients (Table 3). Among the malignant diseases, hematological malignancies were the most frequently observed, and in this study, they were detected in 20 (16.8%) patients; among them, 10 (50%) patients had acute myeloid leukemia, which was mostly detected in CAPA patients [11 (17.5%); *p* < 0.041; Table 3]. The results were confirmed by fiberoptic bronchoscopy with BALF and microscopic examination in 17 (14.3%) patients. Histopathological diagnosis was confirmed in two patients based on the presence of thick, branched fungal hyphae invading the surrounding tissue and causing necrosis, and angioinvasion in the tissue sections was detected by staining with hematoxylin–eosin, periodic acid–Schiff stain, and Grocott-Gomori’s methenamine silver stain (Figure 5). Direct microscopic examination of the sputum or BALF specimens was performed in 17 (14.3%) patients, and the findings confirmed the presence of infection in both the study groups [CAPA (8, 12.7%) vs. CAC (9, 16.1%)]. Serology testing was done in the majority of cases, with IgM and IgG antibodies mostly found in CAPA patients [52 (82.5%)], followed by CAC patients [11 (19.6%); *p* < 0.001]. Galactomannan and mannan tests were performed in 53 (44.5%) patients, and both groups showed similar positive results (*p* < 0.277). The compound (1–3)-β-D glucan was found to be present in 21 (17.6%) patients, but significantly positive results were observed in CAPA patients [16 (25.4%)] when compared to CAC patients [5 (8.9%)] (*p* < 0.019). Blood cultures were positive in 45 (37.8%) patients, with CAC patients being more positive than CAPA patients [40 (71.4%) vs. 5 (7.9%)] (*p* < 0.001). Oxygen support was provided to all the patients, while oxygen masks were used in 65 (54.6%) cases, more often in CAPA than in CAC patients (*p* < 0.015). Mechanical ventilation was applied to 41 (34.5%) patients, while a non-invasive procedure, such as continuous positive airway pressure (CPAP) or high-flow nasal cannula (HFNC), was performed in 20 (16.8%) patients (Table 1). The average duration of mechanical ventilation was almost identical in both the groups, that is, 4 (3–6) days in CAPA and 5 (3–9) days in CAC. The most frequently observed complications during COVID-19 treatment were pneumothorax in 17 (14.3%) patients, pulmonary embolism in 8 (6.7%) patients, spontaneous retroperitoneal hematomas and deep venous thrombosis in 3 (2.5%) patients each (Table 1). Antifungal medications included echinocandins in 42 (35.3%), voriconazole in 30 (25.2%), and fluconazole in 27 (22.7%) patients. In patients treated with voriconazole, the initial dosage was 6 mg/kg twice a day for 2 days, followed by 4 mg/kg twice a day for 14 days, and continued with a maintenance dose of 200 mg/day itraconazole for the next 4–10 weeks. The initial dosage of caspofungin was 70 mg/day and continued at 50 mg/day for 14 days and followed by a Maintenance itraconazole dose was 2x200mg daily at least 6 to 9 months. Overall, all the patients received systemic corticosteroids (mainly methylprednisolone). In addition, 11 patients (9.16%) received favipiravir, 32 (26.67%) received remdesivir, 8 (6.67%) received casirivimab/imdevimab, and 5 (4.16%) received sotrovimab (Table 1). Tocilizumab, an anti-IL-6 agent, was not administered according to the current guidelines. A lethal outcome was observed in 76 (63.9%) patients, which was more frequent in CAC than in CAPA patients (*p* < 0.001).

**Table 1:** Characteristics of patients.

	Total			
	(*N* = 119)	CAPA (*N* = 63)	CAC (*N* = 56)	*p* value
Age	65.7 ± 13.9	61.4 ± 14.7	70.5 ± 11.4	<0.001*
Sex (men)	78 (65.5%)	47 (74.6%)	31 (55.4%)	0.027*
Microscopy	17 (14.3%)	8 (12.7%)	9 (16.1%)	0.600
Blood cultures	45 (37.8%)	5 (7.9%)	40 (71.4%)	<0.001*
Serology	63 (52.9%)	52 (82.5%)	11 (19.6%)	<0.001*
IgM Ab	72 (60.5%)	56 (88.9%)	16 (28.6%)	<0.001*
IgG Ab	53 (44.5%)	31 (49.2%)	22 (39.3%)	0.277
GM	21 (17.6%)	16 (25.4%)	5 (8.9%)	0.019*
BetaDG				
Oxygen mask	65 (54.6%)		24 (42.9%)	0.015*
MV	41 (34.5%)	41 (65.1%)	23 (41.1%)	0.152 1.000
CPAP	7 (5.9%)	18 (28.6%)	3 (5.4%)	0.511
HFNC	13 (10.9%)	4 (6.3%)	5 (8.9%)	8 (12.7%)
Antifungals				
Voriconazole	30 (25.2%)	23 (36.5%)	7 (12.5%)	0.003*
Echinocandins	42 (35.3%)	17 (27%)	25 (44.6%)	0.044*
Fluconazol	27 (22.7%)	12 (19%)	15 (26.8%)	0.314
COVID-19 treatment				
Remdesivir	32 (26.9%)	17 (26.9%)	15 (26.8%)	0.765
Favipiravir	11 (9.2%)	5 (7.9%)	6 (10.7%)	0.015
Casir./Imdevib.	8 (6.7%)	4 (6.4%)	4 (7.1%)	0.145
Sotrovimab	5 (4.2%)	3 (4.7%)	2 (3.6%)	0.123
Complications				
PNTH	17 (14.3%)	8 (12.7%)	9 (16.1%)	0.600
PE	8 (6.7%)	5 (7.9%)	3 (5.4%)	0.721
Haematoma	3 (2.5%)	1 (1.6%)	2 (3.6%)	0.601
DVT	3 (2.5%)	2 (3.2%)	1 (1.8%)	1.000
Died	76 (63.9%)	30 (47.6%)	46 (82.1%)	<0.001*

CAPA, COVID-19-associated pulmonary aspergillosis; CAC, COVID-19-associated candidiasis; IgM Ab, IgM antibodies; IgG Ab, IgG antibodies; GM, galactomannan; Beta DG, beta-D glucan; MV, mechanical ventilation; CPAC, continuous positive airway pressure, HF, high-flow nasal cannula; Casir/Imdevib, casirivimab/imdevimab; PNTH, pneumothorax; PE, pulmonary embolism; DVT, deep venous thrombosis; *Statistical significant importance.

**Table 2:** Serum biomarkers in CAPA and CAC patients.

	Total			
	(*N* = 119)	CAPA (*N* = 63)	CAC (*N* = 56)	*p* value
CRP (mg/L)	112.3 (89)	115.75 (112.5)	113.7 (104.6)	0.869
WBC (10^9^/L)	11 (9)	12.25 (12.8)	11.7 (10.2)	0.175
Ly (10^9^/L)	0.56 (0.84)	0.36 (0.38)	0.47 (0.63)	0.021*
D dimer (μg/L)	2.1 (3.16)	2.26 (2.05)	2.11 (2.7)	0.525
Ferritin (μg/L)	1231 (2052.8)	1965.5 (2899)	1727 (2298)	0.107
LDH (U/L)	915 (512)	873 (637)	876 (600)	0.383
IL 6 (pg/mL)	22.9 (130.9)	120.5 (314.35)	37.7 (248.1)	0.004*

CAPA, COVID-19-associated pulmonary aspergillosis; CAC, COVID-19-associated candidiasis; CRP, C-reactive protein; WBC, white blood cells; Ly, lymphocyte; LDH, lactate dehydrogenase; IL-6, interleukin 6; *Statistical significant importance.

**Table 3:** Comorbidities in CAPA and CAC patients.

	Total	CAPA	CAC (*N* = 56)	*p* value
	(*N* = 119)	(*N* = 63)		
Non-malignant				
HTA	62 (52.1%)	29 (46%)	33 (58.9%)	0.160
DM	34 (28.6%)	18 (28.6%)	16 (28.6%)	1.000
COPD	16 (13.4%)	5 (7.9%)	11 (19.6%)	0.062
CRI	13 (10.9%)	6 (9.5%)	7 (12.5%)	0.603
CMP	9 (7.6%)	4 (6.3%)	5 (8.9%)	0.733
AF	7 (5.9%)	1 (1.6%)	6 (10.7%)	0.051*
CVI	6 (5%)	4 (6.3%)	2 (3.6%)	0.683
Hypothyreosis	6 (5%)	2 (3.2%)	4 (7.1%)	0.418
ARI	4 (3.4%)	2 (3.2%)	2 (3.6%)	1.000
Asthma	4 (3.4%)	4 (6.3%)	0 (0%)	0.121
AC bypass	3 (2.5%)	1 (1.6%)	2 (3.6%)	0.601
PCI	3 (2.5%)	2 (3.2%)	1 (1.8%)	1.000
M.Parkinson	3 (2.5%)	0 (0%)	3 (5.4%)	0.101
Sch	3 (2.5%)	(1.6%)	2 (3.6%)	0.601
Hyperthiresosis	2 (1.7%)	0 (0%)	2 (3.6%)	0.219
Malignant				
Leukemia	14 (11.8%)	11 (17.5%)	3 (5.4%)	0.041*
Solid organ carcin	8 (6.7%)	4 (6.3%)	4 (7.1%)	1.000
Lymphoma	6 (5%)	4 (6.3%)	2 (3.6%)	0.683

CAPA, COVID-19-associated pulmonary aspergillosis; CAC, COVID-19-associated candidiasis; HTA, arterial hypertension; DM, diabetes mellitus; COPD, chronic obstructive pulmonary disease; CRI, chronic renal insufficiency; CMP, cardiomyopathia; AF, atrial fibrillation; PCI, percutaneous coronary intervention; CVI, cerebrovascular insult; ARI, acute renal insufficiency; AC bypass, aortocoronary bypass; M.Parkinson, Morbus Parkinson; Sch, schizophrenia; Solid organ carcin, solid organ carcinoma; *Statistical significant importance.

## Discussion

A total of 120 COVID-19 cases with a proven diagnosis of IFD, who were treated at the COVID Hospital “Batajnica,” Belgrade, Serbia between 1 September and 24 December 2021, were included in this study. The incidence rate of IFD was found to be 1.86% in the 6,335 observed patients. A higher prevalence rate of IFD was detected in men than in women. This finding corresponds with the results of some meta-analysis studies which reported that the risk of severe COVID-19 infection is higher in men than in women (14).

The prevalence rate of IFD in COVID-19 patients, including CAPA and CAC, range from 0.7 to 23.5% depending on the analyzed population (15). In our study, of all the COVID-19 admissions, the prevalence of CAPA in COVID-19 patients was 9.94% (63/6,335), while it was 8.83% (56/6,335) in CAC patients, which was higher than that reported in a recently published study. This outcome is probably because the majority of our patients were hospitalized in ICU (102/120, 85%) (16).

COVID-19-associated mucormycosis (CAM) was reported in the COVID-19 infections with the Delta variant, but this disease was not found in our study group. Decreased immune-cell response in patients with COVID-19 is accompanied by defective lymphocyte count. Severe lymphopenia has been established as a major predisposing risk factor for invasive fungal disease (17). In our study, lymphopenia was more common in CAPA than in CAC patients. In severe cases of COVID-19, particularly those with acidosis, increased levels of lactate acid in the blood and tissue together with lymphopenia can cause IFD (18). A recent study found that critically ill COVID-19 patients with IFD have imbalanced iron levels (19). Hyperferritinemia was also noticed in our study cohort and was associated with serious organ damage, which increases the susceptibility of COVID-19 patients to fungal coinfections (20). Owing to lower blood pH and increased levels of pCO_2_ in COVID-19 patients, ferritin is unstable and tends to release iron, which is further absorbed by fungi to cause infection. The persistence of iron deficiency 2 months after recovery from the COVID-19 disease predisposes COVID-19 patients to a high risk of fungal disease for a prolonged period (21). Most of the patients were admitted to ICU [102 (85%)], mainly because of ARDS and the need for invasive or non-invasive ventilation. Moreover, one-third of the patients [41 (34.5%)] were mechanically ventilated for a median duration of 4–5 days. A total of 18 patients were on non-invasive ventilation (NIV), indicating that the incidence of IFD was higher in patients on MV. Only five patients (12.2%) among the eight intubated ones were satisfactorily extubated (one each with hematological malignancy and COPD and three without comorbidities). Of the 18 patients on NIV, 5 (25%) died. The majority of our patients [71 (59.75%)] had chronic cardiovascular diseases, that is, arterial hypertension in 62 (52.15) and cardiomyopathy in 9 (7.6%) patients, which is one of the fundamental differences between IAPA and IFD, demonstrating that IFD in COVID-19 patients is more common among patients with chronic cardiovascular or pulmonary diseases (7, 22). However, hematologic malignancies were found in 20 (16.7%) patients and other solid malignancies in 8 (6.7%) patients, as reported in a previous publication (23). Bronchoscopy has played a small role in the diagnosis of COVID-19 patients as it is associated with aerosol generation and has a high risk of viral transmission (24). In our study, out of 17 patients, bronchoscopy was performed as a lifesaving procedure because of “*de novo”* atelectasis in 12 (70.6%) patients in ICU and severe hemoptysis in the remaining 5 (29.4%) patients. In 7 out of 17 (41.17%) patients, endoscopy findings have revealed ulcerations, pseudomembranes, and plaque lesions suggestive of CAPA tracheobronchitis. Histopathological confirmation was done only in 2 out of 17 (11.7%) patients showing invasive fungal growth in the tissue, while direct microscopy was done in all the cases, that is, eight (12.7%) in CAPA patients and nine (16.1%) in CAC patients. The detection of galactomannan or mannan in bronchoalveolar lavage fluid is highly indicative of IFD because antigens are released during invasive fungal growth. In patients with severe COVID-19, the detection of galactomannan or mannan in bronchoalveolar fluid is one of the main diagnostic tests for IFD (25). Serum biomarkers show a low sensitivity for galactomannan ranging between 0 and 40% and for (1-3)-β-D glucan ranging between 0 and 50%. Circulating galactomannan is associated with angioinvasion, which increases the likelihood of developing IFD, but in some cases of proven IFD, the serum galactomannan level has been reported to be negative (26). In our study, serum galactomannan antigen was positive in 53 (44.5%) patients. Serum (1–3)-β-D glucan shows higher sensitivity than serum galactomannan, but the only limitation is that (1–3)-β-D glucan is a pan-fungal marker specific for various IFDs. According to the data from a previous study, two consecutive positive results for serum (1–3)-β-D glucan generate a specificity of 90% for IFDs (27). In addition to the serum biomarkers, the blood samples were also used to detect fungal infections based on their growth on a solid culture medium in a large number of our patients [45 (37.8%)], which was more evident in CAC patients. This finding suggests that, during the observed period, when there was a rapid surge in the Delta variant of SARS-CoV2, clinical presentations were extremely challenging. During the study period Delta variant of SARS-CoV-2 became dominant with greater contagiousness, risk for hospitalization and admission in the ICU (28). The diagnostic investigations also include a thorax computed tomography (CT) scan. One of the major difficulties encountered in COVID-19 patients during the late stage of the COVID-19 pandemic was that the disease could be misinterpreted with other superinfections, such as IFDs. Atypical findings in COVID-19 patients suggestive of IFD are multiple pulmonary nodules with cavitations or lobar and segmental infiltrations with partial cavitations (29). Lung imaging findings should be accompanied by samples obtained from the lower respiratory tract, particularly in patients without clinical response or with progressive nodular infiltrates. CT-guided lung biopsy or bronchoscopy with biopsy in patients with severe forms of COVID-19 with suspected IFD should be considered (30). Similar to IAPA patients, *Aspergillus flavus* was the most common pathogen in the CAPA patients involved in our study [53 (84.1%)], while *Candida glabrata* was predominant in CAC patients [20 (35.7%)] (Table 4) (6). In some countries, candidemia was the predominant fungal infection in 87.5% of the patients. *C. albicans* and *C. glabrata* were detected in 14.3% of the cases, and the mortality rate of those infected with either *C. albicans* or *C. glabrata* was 100% even after treatment with appropriate antifungal drugs (31). On the contrary, in our study, non-*Candida albicans* specimens, such as *C. glabrata* and *C. parapsilosis*, were identified more often, that is, in 36 (64.3%) patients (Table 4). Factors that predispose COVID-19 patients to candidiasis are prolonged stays at the hospital or ICU, prone position, medical interventions such as the use of MV and intravenous catheters, and parenteral nutrition (32). A recent study found a direct correlation between the prolonged use of antibiotics, such as ceftriaxone and azithromycin, and the development of candidiasis in COVID-19 patients (33). Evidence from previous studies indicates that patients who developed CAPA had previously been treated with broad-spectrum antibiotics, piperacillin/tazobactam, and meropenem (34). To avoid the possibility of candidemia in the ICU, some authors recommend the use of peripherally inserted central catheters to minimize the risk of contamination by the patient’s oral, nasal, and tracheal secretions during insertion and the use of ultrasound guidance for the insertion of central venous catheters (35). Various complications were noticed during the treatment of COVID-19 patients with IFD [31/120 (25.83%)].

**Table 4:** Pathogens of 120 patients with IFD.

Genus		*N*	%
*Cryotocc* (*N* = 1)	*neoformans*	1	100.0
*Aspergillus* (*N* = 63)	*flavus*	53	84.1
	*fumigatus*	9	14.3
	*niger*	1	1.6
*Candida* (*N* = 56)	*albicans*	2	3.6
	*non albicans*	54	96.4
	*glabrata*	20	35.7
	*parapsilosis*	16	28.6
	*crusei*	6	10.7
	*lusitianiae*	4	7.1
	*laurenti*	3	5.3
	*famata*	3	5.3
	*tropicalis*	2	3.6

Pneumothorax and pneumomediastinum were the most frequent complications [17/120 (14.3%)] without any iatrogenic cause, including mechanical or barotrauma, owing to the widespread alveolar damage in critically ill COVID-19 patients (36). Prothrombotic coagulation abnormalities, including deep venous thrombosis and pulmonary embolism, develop in critically ill COVID-19 patients, which occurred in approximately 16.5 and 14.8% of the patients, respectively, thus contributing to increased morbidity and mortality (37). In our study, pulmonary embolism was noticed in eight (6.7%) patients, while deep venous thrombosis was noticed in three (2.5%) patients.

Bleeding is less commonly present in patients with COVID-19 than thrombosis, but in critically ill COVID-19 patients with IFD, spontaneous intrapulmonary bleeding with hemoptysis and soft tissue hematomas was observed (38). Echinocandins were the most frequently administered first-line antifungal drug therapy for the management of CAC in 42 (35.3%) patients who were either admitted to ICU or were septic shock patients, as reported in a recently published study (16). Triazoles, namely voriconazole and isavuconazole, are recommended as the first-line treatment for IFD (39). Voriconazole is associated with drug–drug interactions in ICU patients and also shows interaction with remdesivir (40). Remdesivir was recommended to 32 (26.67%) patients. In a recent study, posaconazole was shown to be effective in the treatment of IFD (41). Despite the unrestricted use of antifungal drugs in the COVID-19 pandemic, the lethal outcome was noticed in 76 (63.9%) patients, which was significantly more pronounced in CAC than in CAPA patients. Moreover, a higher overall mortality rate due to IFD was detected in patients with COVID-19 when compared to patients without COVID-19 (87.5% vs. 67.9%, respectively) (42).

We can conclude that IFDs play an increasingly important role in COVID-19 patients because they are associated with extensive morbidity and mortality. Ongoing efforts for standardization of diagnostic and therapeutic approaches should be continued. In critically ill COVID-19 patients, clinicians should focus on patient risk factors, such as comorbidities, prolonged stay in ICU, applied ventilation support, and treatment regimen. The impact of corticosteroids and immunobiological therapies on the incidence and mortality of IFD should be taken into consideration while treating COVID-19 patients. Antifungal treatments with prophylactic medications and targeting drugs should also be considered (40, 43).

## Data availability statement

The original contributions presented in the study are included in the article/supplementary material, further inquiries can be directed to the corresponding author.

## Ethics statement

The studies involving human participants were reviewed and approved by Ethics Committee of University Clinical Center of Serbia. Written informed consent from the patients was not required to participate in this study in accordance with the national legislation and the institutional requirements.

## Author contributions

All authors listed have made a substantial, direct, and intellectual contribution to the work and approved it for publication.

## Conflict of interest

The authors declare that the research was conducted in the absence of any commercial or financial relationships that could be construed as a potential conflict of interest.

## Publisher’s note

All claims expressed in this article are solely those of the authors and do not necessarily represent those of their affiliated organizations, or those of the publisher, the editors and the reviewers. Any product that may be evaluated in this article, or claim that may be made by its manufacturer, is not guaranteed or endorsed by the publisher.

## Footnotes

^1^ https://www.R-project.org/
